# When health facilities close, outbreaks follow: the public health consequences of aid cuts in Somalia

**DOI:** 10.1186/s41182-026-01082-7

**Published:** 2026-09-20

**Authors:** Zakaria Abdi Abdullahi, Ismail Mohamud Derow, Mohamed Sharif Abdi, Safia shukri Hilowle

**Affiliations:** 1 Expanded Program on Immunization (EPI) Department, Ministry of Health and Human Services, Mogadishu, Somalia; 2 Research Department, De Martino Hospital, Mogadishu, Somalia; 3 Faculty of Health Sciences, Salaam University, Mogadishu, Somalia

**Keywords:** Somalia, Health facility closures, Humanitarian financing, Aid cuts, Outbreak preparedness, Immunization, Zero-dose children, Malnutrition, Disease surveillance, Public Health

## Abstract

Somalia’s fragile health system is increasingly threatened by reductions in humanitarian financing, with potentially serious consequences for outbreak prevention and public-health preparedness. Recent funding cuts have already resulted in the closure of health and nutrition facilities and substantial reductions in service coverage, with further closures projected under severe funding scenarios. These disruptions extend beyond reduced access to curative care; they may interrupt routine immunization, nutrition screening and treatment, disease surveillance, referral pathways, and early outbreak detection. This is particularly concerning in Somalia, where recurrent measles, cholera, and diphtheria outbreaks occur amid persistent immunization gaps, displacement, conflict, and malnutrition. The 2025 diphtheria resurgence, alongside a substantial population of zero-dose children, demonstrates the risks associated with weak prevention systems. At the same time, the high burden of acute malnutrition increases vulnerability to infectious disease and places additional pressure on already constrained health services. Protecting health facilities should therefore be considered a core outbreak-prevention priority. Sustained financing for primary health care, routine immunization, nutrition, surveillance, and emergency preparedness is essential, particularly in underserved and high-risk communities. Strengthening domestic financing and contingency mechanisms will also be critical to maintaining essential services during future funding shocks and preventing avoidable deterioration in Somalia’s public-health resilience.

## Dear Editor,

When health facilities close in Somalia, the loss is not only clinical capacity but also prevention capacity. In a country where primary care, nutrition services, immunization, and outbreak surveillance remain fragmented and under-resourced, reductions in humanitarian financing risk weakening the infrastructure required to prevent, detect, and respond to infectious disease and nutrition-related emergencies [1].

The urgency is now unmistakable. On 21 August 2026, UNICEF reported that 205 health and nutrition facilities had already closed following unprecedented humanitarian funding cuts, with health and nutrition service coverage reduced by 30%. Under severe funding scenarios, as many as 618 facilities could close nationwide [1]. These closures do not simply reduce access to treatment; they can disrupt vaccination, nutrition screening, referral pathways, disease surveillance, and early outbreak detection. In Somalia, where external financing accounts for a substantial share of health expenditure, abrupt funding reductions can therefore translate into reduced public-health preparedness [2].

This matters because Somalia continues to face recurrent outbreaks in a highly vulnerable setting. WHO identifies conflict, climate shocks, displacement, malnutrition, and infectious disease outbreaks as interconnected drivers of the country’s prolonged health emergency. Recurrent cholera, measles, and diphtheria outbreaks continue to threaten vulnerable populations, while funding constraints place hundreds of health facilities at risk of closure [2]. The 2025 diphtheria resurgence illustrates the consequences of fragile prevention systems: by epidemiological week 33, 1811 suspected cases and 89 deaths had been reported, with 87% of patients having no documented diphtheria immunization [3].

Immunization is particularly vulnerable to service disruption. Somalia continues to have a substantial zero-dose population; recent evidence demonstrates marked regional disparities in childhood immunization coverage, reflecting persistent barriers to access and continuity of services [4]. UNICEF estimated in April 2026 that approximately 700,000 children in Somalia had never received a vaccine, leaving them vulnerable to measles, diphtheria, and polio [5]. Closing facilities serving remote, displaced, and conflict-affected communities could widen these immunity gaps and increase the population susceptible to vaccine-preventable disease.

Malnutrition further amplifies this risk. Nearly 1.88 million children are expected to require treatment for acute malnutrition in Somalia in 2026, including approximately 493,000 with severe acute malnutrition [6].

UNICEF has additionally reported that admissions to stabilization centres increased by 32% during the first six months of 2026, in a context where preventive and community-based nutrition services have been reduced [1]. Malnutrition and infectious disease can reinforce one another, while fragmented access to nutrition, primary care, and referral services may delay early intervention.

The policy implication is clear. Somalia and its partners should protect financing for essential primary healthcare, routine immunization, nutrition, surveillance, and outbreak preparedness, particularly in high-risk and underserved communities. Financing mechanisms should also prioritize integrated service delivery rather than isolated vertical programmes. Over the longer term, stronger domestic financing and contingency mechanisms are needed to prevent essential facilities from closing during droughts, displacement, and outbreaks [2].

Recent analysis further highlights persistent challenges in vaccination coverage, laboratory capacity, surveillance, case detection, and outbreak response, underscoring the need for sustainable financing and stronger prevention systems [7].

Protecting health facilities in Somalia is therefore a core public-health priority, not simply a humanitarian concern. Sustained financing for primary health care, routine immunization, nutrition, surveillance, and outbreak preparedness is essential to prevent further disruption of lifesaving and preventive services. Strengthening domestic financing and contingency mechanisms can help ensure that future funding shocks do not create gaps through which preventable outbreaks can spread.

## Data Availability

No datasets were generated or analysed during the current study.
